# Comparative Analysis of Complications in Early Verses Delayed Laparoscopic Cholecystectomy for Acute Cholecystitis

**DOI:** 10.7759/cureus.78985

**Published:** 2025-02-14

**Authors:** Muhammad Iftikhar, Maria Seereen Qazi, Rashid Khan, Sadeeq Ahmad, Sifat Ullah, Farman Ullah

**Affiliations:** 1 General Surgery, Hayatabad Medical Complex, Peshawar, PAK; 2 General Surgery, Peshawar Institute of Medical Sciences, Peshawar, PAK

**Keywords:** cholecystectomy, complications, early surgery, gall bladder, outcomes

## Abstract

Background: Acute cholecystitis (AC) is one of the most frequent gastrointestinal emergencies, necessitating prompt definitive treatment. Laparoscopic cholecystectomy (LC) has become the gold standard for management, although the timing of surgery remains controversial. Early laparoscopic cholecystectomy (ELC) is performed within 72 hours of symptom onset, while delayed laparoscopic cholecystectomy (DLC) is undertaken after a period of conservative management. The purpose of this study is to assess the complications and outcomes of early vs delayed laparoscopic cholecystectomy in patients with acute cholecystitis.

Objectives: To evaluate perioperative outcomes, including complications, operative time, conversion rates, and hospital stay, between patients undergoing early vs delayed laparoscopic cholecystectomy for acute cholecystitis.

Material and methods: This prospective non-randomized comparative study was conducted in the surgical department of Hayatabad Medical Complex (HMC), Peshawar. The study was carried out during a 15-month period, from June 1, 2023, to August 30, 2024. A total of 118 individuals with acute cholecystitis, age ranged between 18 to 60 years, were included. Acute cholecystitis was diagnosed based on imaging examinations, laboratory tests, and the patient's history.

Results: In Group 1, the mean age of patients was 42.5±10.3 years, and it was 44.1±9.8 years in Group 2. Gender distribution was similar in both groups, with a slight predominance of females (Group 1: 35 females, 25 males; Group 2: 34 females, 24 males). In Group 1, the most common presenting symptoms were right upper quadrant pain (55, 92.4%), nausea (47, 78.8%), and fever (27, 45.8%). Similarly, in Group 2, these symptoms were reported in 53 (91.4%), 46 (79.3%), and 27 (46.6%) patients, respectively. Duration of symptoms prior to surgery was 3.2±1.1 days in Group 1 and 4.1±1.3 days in Group 2.

Conclusions: In comparison to delayed laparoscopic cholecystectomy, early laparoscopic cholecystectomy is associated with fewer complications, fewer readmissions, and shorter hospital stays. These findings have led to the recommendation of early intervention as the best strategy for treating acute cholecystitis.

## Introduction

Acute cholecystitis (AC) is a common gastrointestinal emergency, which is usually treated surgically to avoid complications, such as gangrene, perforation, or sepsis [1]. Laparoscopic cholecystectomy (LC) remains the gold-standard treatment for acute cholecystitis (AC) due to its reduced postoperative pain, faster recovery, and shorter hospital stay. However, there is ongoing debate on the optimal time for laparoscopic cholecystectomy in cases with acute cholecystitis.

Laparoscopic cholecystectomy (LC), indicated for patients with acute cholecystitis within 72 hours, is associated with a lower conversion rate, decreased morbidity, and shorter hospitalization compared to open cholecystectomy [2]. In contrast, delayed laparoscopic cholecystectomy (DLC), done after an initial period of conservative management, has historically been preferentially employed to allow the inflammatory response to resolve, thereby potentially decreasing intraoperative complications [3]. DLC, however, is not without risks, including recurrent attacks, increased probability of complicated disease, and possible challenges in the attempt to adopt a minimally invasive approach [4].

A number of meta-analyses and randomized controlled trials comparing the outcomes of both approaches have produced contradictory results with respect to perioperative morbidity, operative time, and conversion rates [5,6]. Some of them even propose that ELC is associated with lesser overall complications and a greater degree of cost-effectiveness, while others suggest that operating in an inflammatory field increases the risk of bile duct damage [7]. The degree of inflammation of the acute cholecystitis, the patient’s comorbidities, and the level of surgical skill also further dictate the timing of surgery [8].

Despite advances in surgical and perioperative management, there is still controversy regarding the management of patients with acute cholecystitis, particularly in low-resource environments where access to timely surgical intervention is limited [9]. Other factors influencing the decision between early and delayed laparoscopic cholecystectomy, including heterogeneous patient presentation, varying degrees of disease, and institutional guidelines, further complicate clinical practice [10].

The aim of this study is to assess complications with both approaches and make a systematic analysis of the perioperative results of both techniques to provide data on the strategy with the lowest risk and greatest efficacy. These complexities must be considered to translate the findings into the optimization of clinical guidelines, technologies, and, ultimately, patient outcomes, in which clinicians can make personalized decisions based on patient-level needs and local healthcare settings.

## Materials and methods

This prospective comparative study was conducted in the Surgical C unit of Hayatabad Medical Complex (HMC), Peshawar. The study was carried out during a 15-month period, from June 1, 2023, to August 30, 2024. A total of 118 patients with acute cholecystitis were included. Acute cholecystitis was diagnosed based on imaging examinations, laboratory tests, and the patient's history. Clinical signs included fever, leukocytosis, right upper quadrant pain, and a positive Murphy's sign. The gallbladder inflammation was confirmed by imaging studies, including ultrasound and CT scans of the abdomen, which showed the presence of gallstones, thickness of the gallbladder wall, and pericholecystic fluid. All study participants met the following criteria: they were in the target age range, had no prior history of cholecystectomy or biliary system operations, and had been diagnosed with acute cholecystitis. Additionally, the study excluded patients with severe, comorbid conditions that prevented them from undergoing surgery, such as uncontrolled respiratory or cardiac conditions. The study also excluded patients with gangrenous cholecystitis, abscess formation at the time of diagnosis, pregnant and lactating women, and patients with evidence of gallbladder perforation.

Patients were divided into two groups. Patients who underwent surgery within 72 hours after the onset of symptoms were included in the early laparoscopic cholecystectomy (ELC) group and were labeled as Group 1. Patients in the delayed laparoscopic cholecystectomy (DLC) group underwent surgery following conservative treatment, which is typically performed six to twelve weeks after the acute episode ended. Patients were allocated to the ELC or DLC group based on predefined clinical criteria, including symptom duration, severity of inflammation, and patient comorbidities. Surgical scheduling and resource availability were also considered to ensure optimal patient outcomes. After the acute inflammation subsided, the DLC group was conservatively managed with intravenous fluid resuscitation, antibiotic administration, pain control, and laparoscopic cholecystectomy.

Data were collected prospectively using a standardized data collection form to ensure consistency and accuracy. Demographic characteristics, including age, sex, and BMI, were recorded. Duration of symptoms, pain levels, fevers, leukocytosis, and other details of clinical presentation were collected. Operative data is recorded carefully, including the timing of surgery, operative time, conversion to open surgery, and complications during the procedure. Postoperative outcomes, including readmission, mortality, postoperative complications, and length of hospital stay, were also recorded.

Postoperative complications such as bile duct injury, hemorrhage, wound infection, and conversion to open surgery were among the primary outcome measures evaluated. The secondary outcome measures were operative time and length of hospital stay. Statistical analysis was performed using a statistical software program (IBM SPSS Statistics for Windows, Version 25.0. IBM Corp 2017). Continuous variables (age, operative duration) were described as mean±standard deviation (SD), and categorical variables (gender, complication rates) as frequencies and percentages. Continuous variables were compared with the student's t-test, and categorical variables were analyzed using the chi-square test. Statistical significance was defined at p<0.05.

Ethical approval was obtained from the Institutional Review Board (IRB) of Hayatabad Medical Complex, Peshawar (Approval number: 2150). Written informed consent was obtained from all participants, ensuring their understanding of the study’s objectives, procedures, and potential risks.

## Results

Of the 118 patients enrolled, 60 (50.8%) patients underwent early LC, and 58 (49.2%) patients underwent delayed LC. In Group 1, the mean age of patients was 42.5±10.3 years, and it was 44.1±9.8 years in Group 2. Gender distribution was similar in both groups, with a slight predominance of females (Group 1: 35 females, 25 males; Group 2: 34 females, 24 males). In Group 1, the most common presenting symptoms were right upper quadrant pain 55 (92.4%), nausea 47 (78.8%), and fever 27 (45.8%), and in Group 2, these symptoms were present for 53 (91.4%), 46 (79.3%), and 27 (46.6%) of patients, respectively. Duration of symptoms prior to surgery was 3.2±1.1 days in Group 1 and 4.1±1.3 days in Group 2. These results are detailed in Table 1.

**Table 1:** Demographics and other clinical characteristics. The data has been represented as n %,  and Mead±SD.

Characteristic	Group 1	Group 2
Mean age	42.5±10.3	44.1±9.8
Gender (female/male)	35 (58.3%), 25 (41.7%)	34 (58.6%), 24 (41.4%)
Duration of symptoms (days)	3.2±1.1	4.1±1.3
Right upper quadrant pain	55 (92.4%)	53 (91.4%)
Nausea	47 (78.8%)	46 (79.3%)
Fever	27 (45.8%)	27 (46.6%)

The mean operative time in Group 1 was 60.2±12.4 minutes, as opposed to 75.6±15.3 minutes in Group 2. The conversion rate to open surgery was lower in Group 1 (3.3%) than in Group 2 (10.3%). Mean blood loss in Group 1 was 45.6±8.2, and it was 52.4±10.1 in Group 2. These results are detailed in Table 2. 

**Table 2:** Operative findings. The data has been represented as n % and Mead±SD. Statistical test for operative time and blood loss is  t-test; conversion to open surgery is done by chi-square test.

Outcome	Group 1	Group 2	p-value
Operative time (minutes)	60.2±12.4	75.6±15.3	<0.001
Blood loss (mL)	45.6±8.2	52.4±10.1	<0.001
Conversion to open surgery	2 (3.3%)	6 (10.3%)	0.251

The overall complication rate was lower in Group 1 (10%) than in Group 2 (27.6%). In Group 1, a postoperative bile duct injury was recorded in 1.7% of cases, wound infection in 3.3% of cases, an intra-abdominal abscess in 5% of cases, and postoperative hemorrhage and urinary retention in 1.7% of cases. The mean length of hospital stays was 4.2±1.3 days in Group 1 and 6.5±2.0 days in Group 2. These outcomes are shown in Table 3.

**Table 3:** Outcome of the study. The data has been represented as n %  and Mead±SD. The statistical test used are t-test and chi-square test.

Complication	Group 1 (n=60)	Group 2 (n=58)	p-value	χ² value/t-test value
Total complications (%)	6 (10%)	16 (27.6%)	0.027	4.917
Bile duct injury (%)	1 (1.7%)	4 (6.9%)	0.346	0.889
Wound infection (%)	2 (3.3%)	6 (10.3%)	0.251	1.32
Intra-abdominal abscess (%)	3 (5.0%)	8 (13.8%)	0.185	1.76
Postoperative hemorrhage (%)	1 (1.7%)	2 (3.4%)	0.999	0
Urinary retention (%)	1 (1.7%)	3 (5.2%)	0.59	0.291
Length of hospital stay (days)	4.2 ± 1.3	6.5 ± 2.0	<0.001	-7.431 (t-test)

## Discussion

The treatment of acute cholecystitis remains a contentious issue in surgical practice, especially with regard to the timing of LC [11]. The results of this study indicate that early laparoscopic cholecystectomy reduces the risk of complications, shortens operative time, and reduces hospital stay compared to delayed laparoscopic cholecystectomy.

The overall complication rate was lower in Group 1 (10%) compared to Group 2 (27.6%), which is in line with the reported findings of Papi et al., who observed early LC resulted in fewer complications compared to those who underwent delayed surgery [12]. Ambe et al. study showed that early LC and delayed LC were associated with similar rates of complications and that individual patient characteristics and the surgeon’s experience may affect the results [13]. Such contradictions indicated opportunities for improvement in the selection of patients and the timing of surgical treatment in relation to clinical features.

The average operative time observed in Group 1 was 60.2±12.4 minutes. This was significantly shorter than the operative time observed in Group 2, which was 75.6±15.3 minutes. Such conclusions are in line with a study by Song et al., who reported that early LC was associated with a shorter duration of surgery because the surgeries involved less severe inflammation and easier dissection [14]. By contrast, a study by Sanchez et al. reported no significant difference in the operative times of either group and postulated that the difference could be due to the level of experience of the surgeons [15].

The early LC group (Group 1) recorded fewer incidences of bile duct injuries (1.7%) than Group 2 (6.9%). This corroborates the findings of Skouras et al., highlighting the protective role of early surgical intervention on the biliary tree [16]. The research conducted by Macafee et al. considered that iatrogenic complications could be more prevalent if less experienced surgeons attempted early surgery, recommending that early surgery should only be performed by experienced surgeons [17].

In this study, the data on postoperative complications also demonstrated the benefits of early LC. The rates recorded for wound infection were 3.3% in Group 1 and 10.3% in Group 2, and findings in both groups were in line with the findings of Yamashita et al., who reported a sharp decrease in the rates of postoperative complication after early LC [18]. Conversely, Casillas et al. reported no differences in the infection rates in the two groups, suggesting that other factors, such as perioperative antibiotic use and other comorbidities, might have a significant impact [19].

The duration of hospital stays was markedly lower in Group 1 (4.2±1.3 days) than in Group 2 (6.5±2.0 days, p<0.001), consistent with results from the Gurusamy et al. study on clinical and economic advantages of early intervention [20]. A different study by Cao et al. did not establish substantial differences in the hospital stay durations that would indicate effective recovery after surgery, and thus, the relevance of timing to the recovery may not be absolute [21]. Also the conversion to open surgery was lower in the ELC group (3.3%) vs the DLC group (10.3%) but not statistically significant. This aligns with the literature that early intervention reduces the chance of severe inflammation or anatomical distortions that require open surgery. The less blood loss in the ELC group (45.6±8.2 ml vs 52.4±10.1 ml) supports the technical advantage of operating in the early phase of the disease.

The study's strengths include a well-defined patient group that increases the reliability of the results, a prospective design, and standardized data collection. The study provides important information on the advantages of early intervention by comparing the intraoperative and postoperative results of early and delayed laparoscopic cholecystectomy. Because the trial was conducted at a busy surgical center with skilled surgeons, the findings demonstrate strong clinical outcomes and a high standard of care. However, there are some limitations to consider. Non-randomized design may introduce selection bias since surgery timing is determined by clinical judgment rather than randomization. The study was conducted at a single tertiary care hospital, which may not be generalizable to other populations or healthcare systems. Future randomized control trials with multicenter institutions and longer follow-up periods are recommended. This will help to validate the results and determine the optimal timing for laparoscopic cholecystectomy.

## Conclusions

Early laparoscopic cholecystectomy (ELC) performed within the first 72 hours of diagnosis of acute cholecystitis lowers complication rates and reduces procedure duration and even the time taken for hospitalization when compared with delayed laparoscopic cholecystectomy (DLC). This fits the findings and recommends the replacement of the current approach with ELC, which is consistent with the current trends on the need for surgery at the earliest possible time. Achieving the aim in prospective studies with minimal complications will improve the overall management of the condition. This study suggests such steps would be best addressed through the implementation of more randomized control trials in the future.
